# Development and validation of a nomogram for predicting the 6-months survival rate of patients undergoing incident hemodialysis in China

**DOI:** 10.1186/s12882-022-02864-x

**Published:** 2022-07-01

**Authors:** Guode Li, linsen Jiang, Jiangpeng Li, Huaying Shen, Shan Jiang, Han Ouyang, Kai Song

**Affiliations:** 1 Department of Cardiology, Maoming People’s Hospital, Maoming, Guangdong China; 2 Department of Nephrology, The Second Affiliated Hospital of Soochow University, 1055San-Xiang Road, Suzhou, 215004 Jiangsu Province China

**Keywords:** Hemodialysis, Nomogram, Prediction model, Survival, All-cause mortality

## Abstract

**Background:**

The all-cause mortality of patients undergoing hemodialysis (HD) is higher than in the general population. The first 6 months after dialysis are important for new patients. The aim of this study was to develop and validate a nomogram for predicting the 6-month survival rate of HD patients.

**Methods:**

A prediction model was constructed using a training cohort of 679 HD patients. Multivariate Cox regression analyses were performed to identify predictive factors. The identified factors were used to establish a nomogram. The performance of the nomogram was assessed using the C-index and calibration plots. The nomogram was validated by performing discrimination and calibration tests on an additional cohort of 173 HD patients.

**Results:**

During a follow-up period of six months, 47 and 16 deaths occurred in the training cohort and validation cohort, respectively, representing a mortality rate of 7.3% and 9.2%, respectively. The nomogram comprised five commonly available predictors: age, temporary dialysis catheter, intradialytic hypotension, use of ACEi or ARB, and use of loop diuretics. The nomogram showed good discrimination in the training cohort [C-index 0.775(0.693–0.857)] and validation cohort [C-index 0.758(0.677–0.836)], as well as good calibration, indicating that the performance of the nomogram was good. The total score point was then divided into two risk classifications: low risk (0–90 points) and high risk (≥ 91 points). Further analysis showed that all-cause mortality was significantly different between the high-risk group and the low-risk group.

**Conclusions:**

The constructed nomogram accurately predicted the 6-month survival rate of HD patients, and thus it can be used in clinical decision-making.

## Introduction

The mortality of patients undergoing dialysis is relatively high despite the large amount of resources directed towards the treatment of end-stage renal disease (ESRD). Specifically, the all-cause mortality of patients undergoing dialysis is about seven times higher compared with that of the general population [1]. A previous study found that the mortality of hemodialysis (HD) patients was higher in the first year, especially within the first three months after dialysis [2, 3]. Data from European and American national databases have revealed that the mortality of HD patients within 90 days after initiation of dialysis ranged between 5.6% and 8.6%, whereas the mortality within one year was between 16.2% and 24.3% [2]. Furthermore, patients who died within 90 days after dialysis accounted for 35% to 50% of deaths within one year [4].

The first three months after initiating dialysis represent an important transitional period for new dialysis patients. Studies have reported that early death of HD patients is often defined as death within three months after the beginning of dialysis [5]. However, only few studies have focused on the mortality of HD patients within six months. A study by Lewis et al. [6] developed and validated an integrated 6-month prognostic tool to monitor the progress of HD patients, however, they did not include common hematologic indicators and drugs used by HD patients. Even though Santos et al. [7] used readily available clinical information to derive and internally validate a 5-variable tool for predicting the 6-month mortality among older adults after undergoing dialysis, their findings cannot be generalized to the HD population given that only elderly patients were enrolled in the study. Although early mortality in HD patients is high and seriously affects the prognosis of patients, better and more accurate clinical risk prediction models for predicting the 6-month survival rate are lacking.

Risk prediction model, a mathematical model for predicting the probability of end-point events, has been widely used in the medical field, as in the EuroSCORE II model for predicting the risk of heart surgery and the Charlson Comorborbidity Index (CCI) for predicting survival of cancer patients [8, 9]. Nomograms have been shown to be effective in predicting all-cause mortality or cardiovascular mortality in dialysis patients [10, 11]. Herein, we aimed at developing and validating an easy-to-use nomogram for predicting the 6-month survival rate of patients undergoing HD.

## Patients

A total of 679 adult HD patients were enrolled at the Second Affiliated Hospital of Soochow University in China from 31^st^ January 2009 to 31^st^ December 2013. The exclusion criteria were: (1) under the age of 18 years; (2) history of kidney transplantation; (3) chronic peritoneal dialysis (PD); and (4) comorbid with malignant tumor. A total of 643 adult incident HD patients met the inclusion criteria and were assigned to the testing cohort. In addition, a validation cohort comprising 173 adult ESRD patients who underwent dialysis at another independent dialysis center between 31^st^ January 2016 and 31^st^ May 2020 were included. The study protocol was approved by the Clinical Research Ethics Committee of The Second Affiliated Hospital of Soochow University and is registered in the Chinese Clinical Trial Registry (NO. ChiCTR 1,900,024,999). All participants provided signed informed consent to participate prior to the study.

### Clinical and laboratory parameters

The following laboratory parameters were recorded: creatinine (Cr), hemoglobin (HB), albumin (Alb), blood urea nitrogen (BUN), serum uric acid (UA), calcium (Ca), phosphorus (P), potassium (K), low-density lipoprotein cholesterol (LDL), total triglycerides (TG), total cholesterol (TC), parathyroid hormone (PTH), high-sensitivity C-reactive protein (Hs-CRP), total Kt/V, and estimated glomerular filtration rate (eGFR). The Kt/V results were obtained using the second-generation Daugirdas formula (Kt/V_Dau_) [12] which has been verified and proved to be one of the most accurate Kt/ V formulas [13]. eGFR of HD patients was estimated using the CKD Epidemiology 2009 creatinine equation [14]. The patient’s 24-h urine output was accurately recorded by a nurse during the first hospitalization.

### Candidate variables

Demographic variables such as age, smoking, and gender were included as candidate variables, whereas blood pressure, height, and dialysis dry weight were included as physical examination variables. In addition, concurrent disease, including diabetes, hypertension, cerebrovascular disease, and cardiovascular disease were included. Drug information was defined as patients taking these drugs orally for at least six months before starting dialysis treatment. All information were obtained at or before dialysis initiation. Body mass index (BMI) was calculated according to the height and weight. Hypertension was based on at least two separate blood pressure measurements ≥ 130/80 mmHg. The chronic kidney disease stages were categorized according to the Kidney Disease Outcomes Quality Initiative (KDOQI) HD clinical practice guidelines [15]. Intradialytic hypotension was determined as follows: systolic blood pressure drop by more than 20 mmHg or mean arterial pressure (MAP) drop by more than10 mmHg during dialysis treatment. The associated symptoms of hypotension were also recorded.

### Follow-up and outcome

Patients in the training and validation cohorts were followed for six months after initiation of HD treatment. The outcome of interest was all-cause mortality which was defined as death due to cardiovascular disease, cerebrovascular disease, infectious disease, multiple organ failure, secondary malignant neoplasms, and other reasons. All patients were followed up until death, transfer to PD treatment, undergoing renal transplant, or transfer to another dialysis center.

### Statistical analysis

All statistical analyses were performed using the SPSS software (version 23.0) and R software (version 3.6.2). R Statistical Software with “rms” packages and five predictors (“age”, “temporary dialysis catheter”, “intradialytic hypotension”, “use of ACEi or ARB”, and “use of loop diuretics”) were used for statistical analyses and to establish a nomogram. Points are allotted for each variable by drawing a straight line upward from the corresponding value to the points line. Next, all points were summed up and the number on the total points axis was located. The variables of training and validation datasets with a normal distribution are presented as mean ± SD and compared using t-test. Variables with skewed distribution are presented as medians with interquartile range and compared by the Mann–Whitney U test. Categorical variables are presented as proportions and compared using a *χ*^2^ test. All candidate variables (*p* < 0.30) were subjected to backward elimination for multivariable logistic regression analysis. The backward elimination was started with all candidate predictors after which a sequence of tests was performed to remove or retain variables in the model based on *p* < 0.05. In addition, the hazard ratio and the 95% confidence interval (95% CI) were calculated. *P* < 0.05 was considered statistically significant for all tests.

Multivariable analysis was performed using Cox regression models to develop a nomogram. Next, the predictive performance of the nomogram was evaluated using the C-index. Calibration was performed through bootstrapping with 1000 research resamples and assessed using calibration plots, which measured the relationship between predicted probabilities and observed proportions. Decision curve analysis (DCA) was conducted to determine the clinical usefulness of the survival nomogram by quantifying the net benefits at different threshold probabilities in the cohort. Furthermore, patients were categorized into ‘low’ or ‘high’ risk groups using recursive partitioning tree analysis to generate the optimum cut-off point. Finally, Kaplan–Meier curves were plotted for the two risk groups.

## Results

### Baseline characteristics

**Fig. 1:**
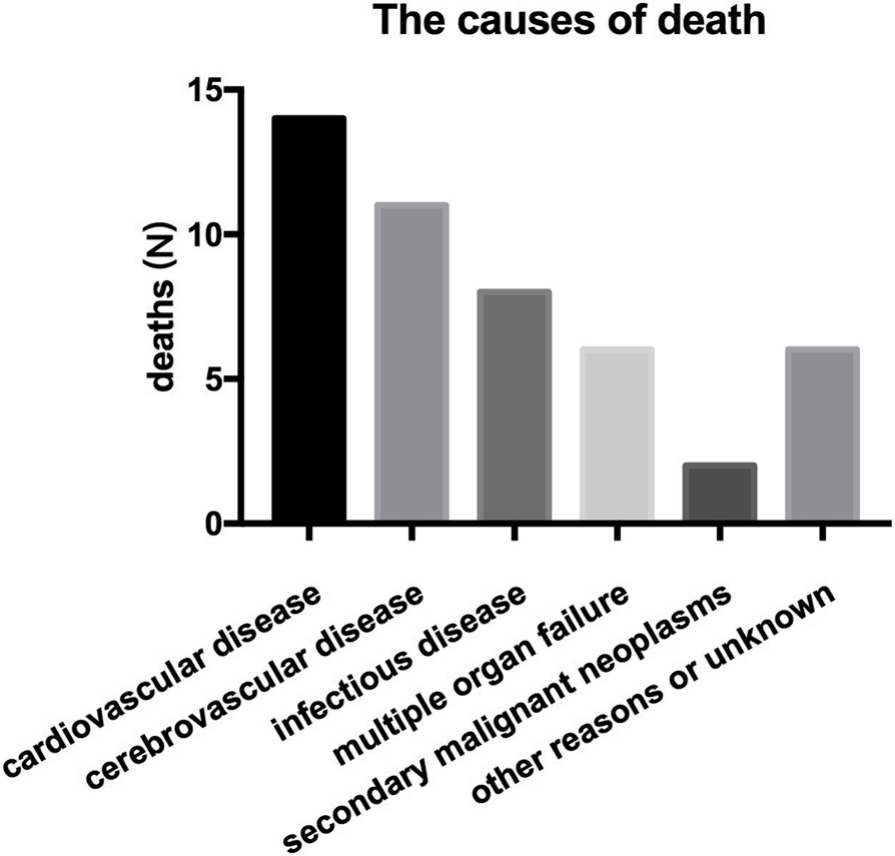
Different causes of death in hemodialysis patients

The baseline characteristics of the two groups recorded at or before dialysis initiation are summarized in Table 1. Patients in the training and validation cohorts had similar demographic characteristics, comorbidities, laboratory data, medicine use, and outcomes (Table 1). During the 6 months follow-up, there were 47 (7.3%) and 16 (9.2%) deaths in the training cohort and validation cohort, respectively. Fourteen (29.79%) deaths in the training cohort were attributed to cardiovascular diseases. Figure 1 shows the detailed causes of death.

**Table 1:** Baseline Characteristics of the study populations and subpopulations

**Characteristic**	Training dataset (*n* = 643)	Validation dataset (*n* = 173)	*P*-value
Age at diagnosis (years), mean ± SD	57.41 ± 15.84	57.50 ± 16.21	0.421
Male	311(48.37)	84(48.55)	0.399
Body mass index (kg/m^2^)	21.49 ± 3.20	21.32 ± 3.13	0.187
Smokers n (%)	76(11.82)	22(12.72)	0.451
Diabetes n (%)	202 (31.42)	66 (32.20)	0.289
Hypertension n (%)	589(91.60)	153(88.44)	0.081
Intradialytic hypotension	195(30.32)	48(27.75)	0.261
Cardiovascular disease n (%)	231(35.93)	65(37.57)	0.687
Systolic blood pressure (mmHg)	142.22 ± 21.31	141.53 ± 22.05	0.573
Diastolic blood pressure (mmHg)	85.24 ± 32.22	83.35 ± 15.71	0.311
Serum creatinine (umol/L)	802.32 ± 353.13	799.32 ± 345.89	0.282
Serum uric acid (umol/L)	418.00(179.75)	413.50(181.25)	0.832
Blood urea nitrogen(BUN)	22.45(14.17)	23.54(13.11)	0.613
Hemoglobin (g/L)	89.39 ± 22.51	88.82 ± 22.12	0.413
White blood cell count(10^9^ /L)	6.34 ± 3.12	6.29 ± 3.51	0.424
Serum albumin (g/L)	33.85 ± 6.80	32.99 ± 6.59	0.829
Serum calcium (mmol/L)	2.09 ± 0.51	2.07 ± 0.21	0.622
Serum phosphorus (mmol/L)	1.89 ± 0.53	1.82 ± 0.62	0.981
Serum potassium(mmol/L)	4.38 ± 0.83	4.56 ± 0.80	0.431
Triglycerides (mmol/L)	1.32(0.92)	1.35(0.90)	0.892
Total cholesterol (mmol/L)	4.18(1.49)	4.22(1.41)	0.792
Low density lipoprotein (mmol/L)	2.28(1.24)	2.35(1.13)	0.594
Hs-CRP (g/mL)	6.50(12.00)	6.60(12.60)	0.813
PTH (pg/ml)	246.35(285.40)	339.12(398.76)	0.252
24 h urine output < 400 ml	263(40.90)	66(38.15)	0.175
Kt/V	1.23 ± 0.32	1.26 ± 0.26	0.441
eGFR (ml/min/1.73m^2^)	5.38(4.05)	5.52(4.21)	0.438
ACEi or ARB n (%)	212(32.97)	56(32.37)	0.539
CCB n (%)	432(67.19)	126(72.83)	0.171
The initial dialysis access			
(1)Use of AVF n (%)	196(30.48)	50(28.90)	0.190
(2)Use of semi-permanent dialysis catheter n (%)	88(13.69)	34(19.65)	0.120
(3)temporary dialysis catheter n (%)	359(55.83)	89(51.45)	0.253

*Abbreviations*: *ACEi* angiotensin-converting enzyme inhibitor, *ARB* angiotensin receptor blocker, *CCB* calcium channel blocker, *Hs-CRP* high-sensitivity C-reactive protein, *PTH* parathyroid hormone, *eGFR* estimated glomerular filtration rate, *AVF* arteriovenous fistula

### Selection of variables

**Fig. 2:**
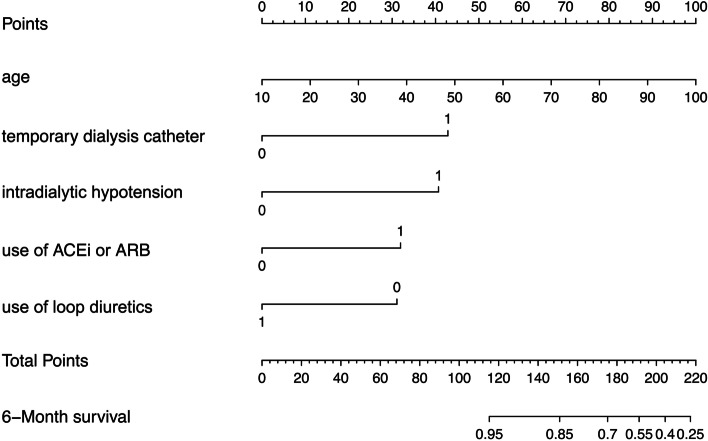
Nomogram for predicting the risk of all-cause mortality in HD patients. For example, an 60-year-old (55 points) HD patient took ACEi (32 points) before dialysis but did not take loop diuretics (32 points). HD was performed using AVF without a history of temporary dialysis catheter (0 point) and with a history of intradialytic hypotension (40 points) at dialysis initiation had a total risk score of 159 points, corresponding to 6-month probabilities of survival of about 80%

In the training cohort, univariate analysis identified eight candidate predictors that were closely associated with the all-cause mortality (Table 1), including “age”, “platelet”, “White blood cells”, “temporary dialysis catheter”, “intradialytic hypotension”, “LDL”, “use of ACEi or ARB”, and “ use of loop diuretics”. After multivariable Cox regressive analysis, five predictors were included in the final multivariable model (Table 2): “age”, “temporary dialysis catheter”, “intradialytic hypotension”, “use of ACEi or ARB”, and “use of loop diuretics”. Finally, the nomogram was used to develop a score for predicting the survival based on these five predictors (Fig. 2).

**Table 2:** Multivariable Hazard Ratios for the Relationship Between Prognostic Risk Factors and 6-Month All-Cause Mortality

*Variable*	*Coeffificient*	*HR ( 95% CI)*	*P*-value
Age	0.031	1.032 (1.009–1.054)	0.005
Intradialytic hypotension	0.611	1.842 (1.159–2.928)	0.010
Use of ACEi or ARB	0.539	1.741 (1.088–2.702)	0.020
Use of loop diuretics	- 1.569	0.208 (0.127–0.342)	0.000
Temporary dialysis catheter	0.686	1.986 (1.294–3.047)	0.002

Univariate analysis identified 8 candidate predictors that were closely associated with the all-cause mortality, including “age”, “platelet”, “White blood cells”, “temporary dialysis catheter”, “intradialytic hypotension”, “LDL”, “use of ACEi or ARB”, and “ use of loop diuretics”. After that, we used multivariable Cox regressive analysis on all-Cause Mortality (after multivariate adjustment for gender, diabetes, hypertension and cardiovascular disease), five predictors (Table 2) were included in the final multivariable model

### Nomogram for predicting survival

Multivariable Cox regression and hazard ratios (HR) were calculated for the prognostic factors used to establish the nomogram (Table 2). In the training cohort, increasing age, temporary dialysis catheter, intradialytic hypotension, use of ACEi or ARB, and use of loop diuretics were found to be associated with survival outcome from all causes after the 6-months follow-up. The linear predictors obtained from the Cox regression model were used to develop a nomogram for predicting survival of HD patients (Fig. 2).

### Validation of the nomogram

**Fig. 3:**
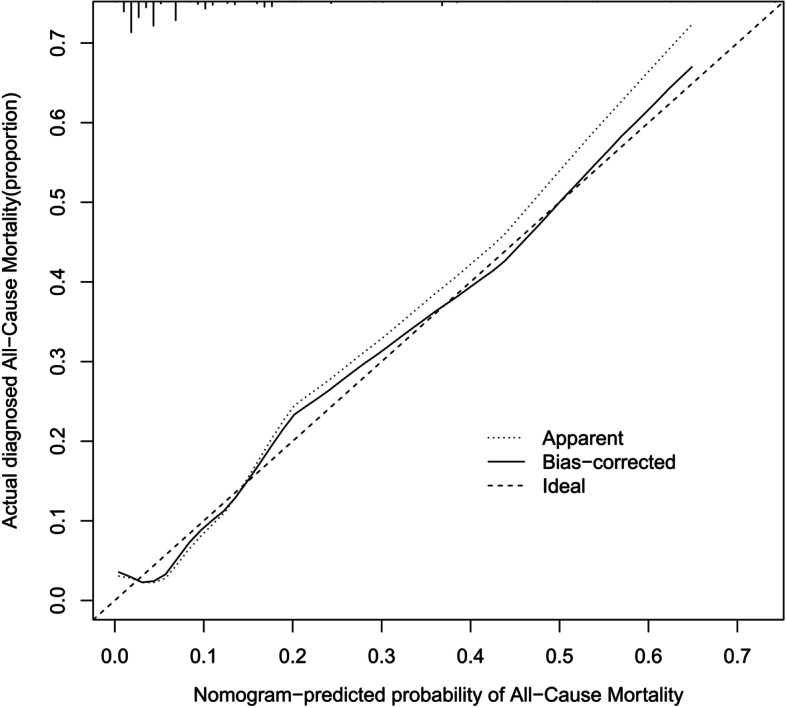
Calibration plots for predicting probability of all-cause mortality in the training cohort. A 45.^0^diagonal line indicates perfect calibration

**Fig. 4:**
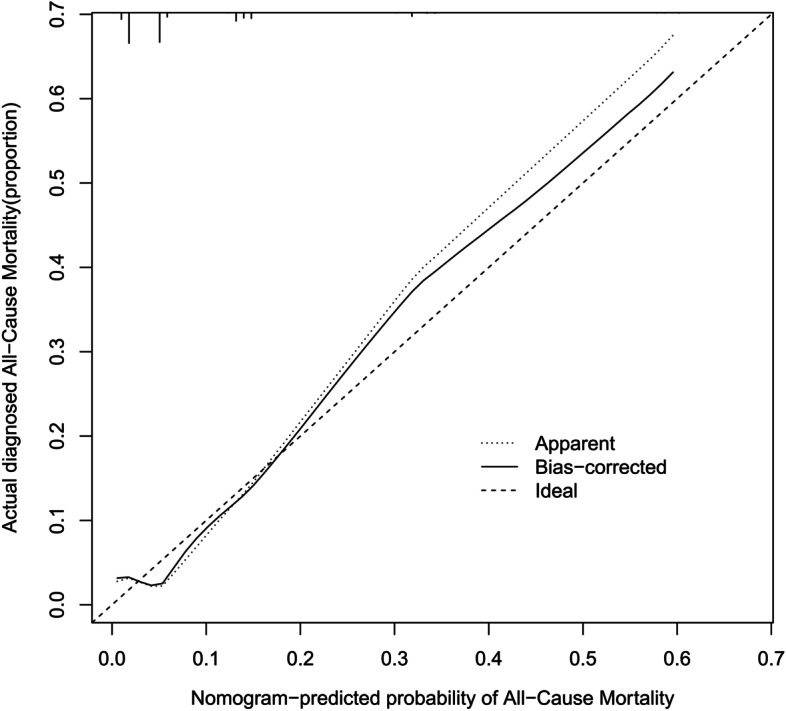
Calibration plots for predicting probability of all-cause mortality in the validation cohort

**Fig. 5:**
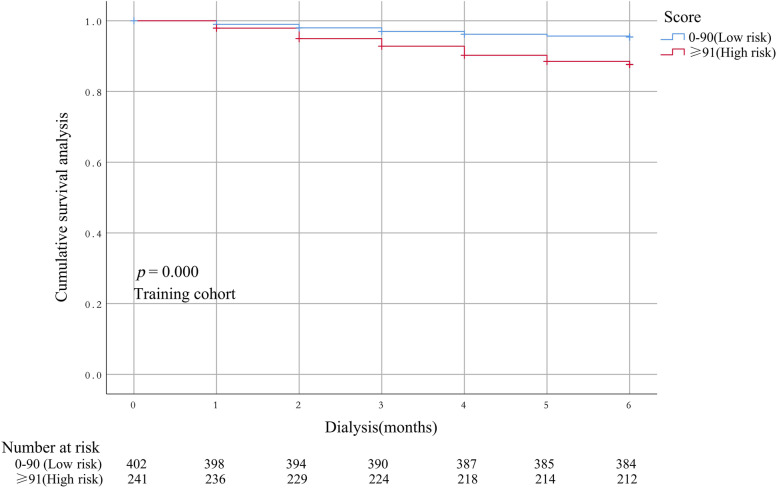
Kaplan–Meier survival curves for the training cohort on the basis of the nomogram

**Fig. 6:**
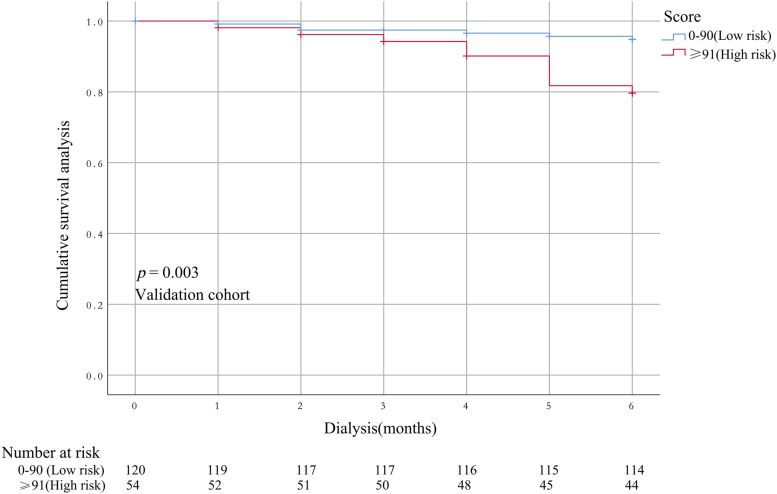
Kaplan–Meier survival curves in the validation cohort on the basis of the nomogram

The performance of the model in the training and validation cohorts was assessed using discrimination and calibration indexes. The score revealed good discrimination in the training cohort [C-index 0.775(0.693–0.857)] and validation cohort [C-index 0.758(0.677–0.836)], and the calibration plots showed good calibration (Figs. 3, 4). The model appeared to be well-calibrated, and showed a good fit between the predicted probabilities and observed proportions. Using the five predictors, the nomogram was adopted to develop a score for predicting the survival probability. The total possible points for the score ranged from 0 to 186 according to the classification and regression tree model. Next, patients were divided into two survival risk groups: low risk (0—90 points) and high risk (≥ 91 points). Finally, Kaplan–Meier curves were plotted for these two risk groups (Figs. 5, 6).

### Clinical utility

**Fig. 7:**
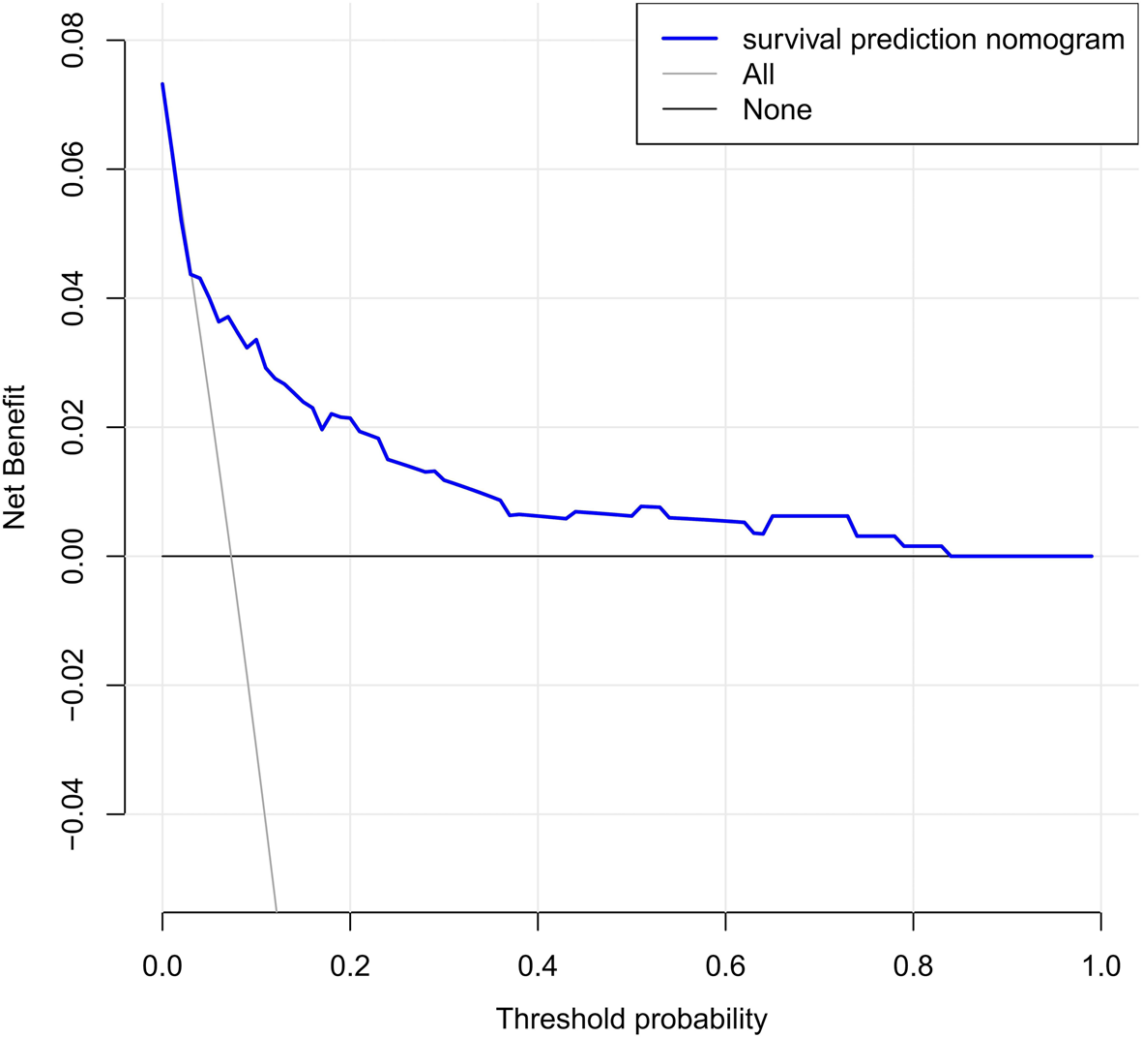
Decision curve analysis for the survival nomogram

The DCA of the nomograms is presented in Fig. 7. The net benefit was calculated by adding the true positives and subtracting the false positives. The straight line represents the assumption that all patients will die, whereas the horizontal line represents the assumption that no patient will die. Results of the DCA demonstrated that the nomogram added more net benefit compared to the treat-all strategy or treat-none strategy with a threshold probability ≥ 5%.

## Discussion

The Dialysis Outcomes and Practice Pattern Study (DOPPS) study conducted in 11 countries showed that the highest mortality of HD patients was observed in the first month after dialysis [16]. It is well documented that the mortality of HD patients is higher within three to six months after initiation of dialysis. According to the United States Renal Data System (USRDS) report [1], all-cause mortality peaked about two months after dialysis initiation in HD patients. Therefore, the high mortality rate of dialysis patients in the early stage of HD should not be ignored. In the present study, we developed and validated a model for predicting all-cause mortality risk among incident HD patients in the first 6-months using five easily available baseline variables.

The five predictors were: age, temporary dialysis catheter, intradialytic hypotension, use of ACEi or ARB, and use of loop diuretics. Traditional risk factors for death and dialysis-related factors were included. The easy and calculable score described here was designed to identify HD patients who were at high risk of death during the first six months after initiation of dialysis. This model can not only identify patient risk factors for early death, but also help health care workers to implement targeted treatment measures for patients. Identifying death risk factors for dialysis patients in early stage can help initiate early interventions for those at risk. Among the risk factors include hypertension and hypotension management, choice of the dialysis access, and strategies for the use of ACEi or ARB or diuretics in different populations.

Multiple studies, including ours, have reported several clinical models for predicting all-cause mortality in HD patients in the past years [17–21]. a numerous mortality scores for dialysis patients have been established on the basis of various comorbidities and laboratory data, but only a few can predict the short-term survival. Therefore, few data are available for developing tools for predicting the risk of early death in HD patients.

Although scoring systems for elderly HD patients have been reported in multiple countries [19–21], these clinical models do not include Asian populations. A prognostic score was developed to predict the 6-month prognosis of elderly patients in French HD patients [19]. In the study, 9 risk factors were identified. Among them, unplanned dialysis overlapped with the temporary dialysis catheter identified in the present study. Other factors were unique to the clinical model (e.g., congestive heart failure, peripheral vascular disease, cancer, and serious functional limitations, BMI, diabetes, arrhythmia, and severe behavioral disorders). However, they found that age was not an independent risk factor of mortality, which differs from our model and other clinical models. Therefore, the application of their model to Chinese HD patients may be limited given the important differences in practice patterns. Thamer et al. [20] developed a clinical score to predict mortality in the first 3 and 6-months based on US Renal Data System comprising 7 predictors. of which “age” is the similar factor to our model. However, the other 6 predictors were not available in our data. Wick et al. [21] utilized a big population-based data source in outpatient settings to develop a score for elderly dialysis patients. Their model for predicting the 6-month mortality included 7 predictors: age (≥ 80 years), increased eGFR, hospitalization in the prior 6 months, atrial fibrillation, congestive heart failure, lymphoma and metastatic cancer, none of those variables except older age were strongly predictive in our model. These three studies could be related to differences in the populations from which they were included (Chinese as opposed to Canadian or American or French). In addition, some factors, such as “cancer” were not included in our inclusion criteria. The risk scores reported by Thamer et al., (AUC = 0.69–0.72), Couchoud et al., (0.68–0.74) and Wick et al., (c-statistic = 0.72) showed fair performance in predicting the risk of early death in HD patients. Compared with these three models, the tool established in the present study showed good discrimination (c-statistic = 0.775).

In another prediction model for predicting early mortality in United Kingdom HD patients reported by Wagner [22] et al., several clinical variables were identified among which two risk factors were used in the presented study (i.e., age and dialysis modality). Our risk prediction model included 3 variables that were not included in previous tools, namely intradialytic hypotension, use of ACEi or ARB, and use of loop diuretics. In this study we found that patients with intradialytic hypotension had a higher mortality compared with those with normal or hypertension in the first six months after initiating dialysis. It has previously been reported that intradialytic hypotension is a common complication of HD patients, which may be associated with decreased blood volume, autonomic nervous dysfunction, cardiac dysfunction, and vascular dysfunction during dialysis [23]. Young et al. found that ACEi and ARB have different efficacy in regulating hemodynamics, cardiovascular remodeling, cardiovascular events, and all-cause death in HD patients [24]. However, in this study, we found that HD patients using ACEi or ARB had a lower 6-month survival rate, which differs from previous studies and may be related to hyperkalemia. Movilli et al. reported that ACEi/ARB treatment increased the risk of hyperkalemia in anuric HD patients suggesting that great caution should be applied in the wider utilization of this class of drugs in anuric HD patients [25]. In a 3-year study of 74,000 HD patients, Sanghavi et al. found that a pre-dialysis serum potassium concentration of more than 6 mEq/l was associated with 50% higher risk of cardiovascular mortality and all-cause mortality [26]. These results suggest that hyperkalemia caused by ACEi/ARB or other factors may be risk factors of death in HD patients. However, the effect of ACEi/ARB on HD patients is still controversial which need to be clarified in future clinical studies. It has been reported that continued use of loop diuretics during the first year of dialysis is associated with lower hospitalization rates, lower intradialytic hypotension rates, and lower interdialysis weight gain, but does not affect mortality [27]. Herein, the results showed that use of loop diuretics before dialysis initiation reduced the risk of death within the first six months. In addition to increasing urine output, loop diuretics improve sodium excretion by about 20% and is unaffected by the levels of eGFR in different types of kidney disease [28], similarly, use of diuretics was shown to increase urine volume, sodium and potassium excretion in dialysis patients [29] and Bragg-Gresham et al. reported that volume managed with diuretics had a 7% lower all-cause mortality risk and 14% lower cardiac-specific mortality risk in HD patients which is similar to our finding [30].

Our clinical model has a few strengths. Firstly, to our knowledge, this is the first prognostic score for predicting early death (within 6 months) in HD patients that is developed and externally validated in a Chinese population. In some previous studies, the models were only internally validated. Second, the constructed nomogram is simple, practical, and robust, all the variables can be collected easily and the risk of early death can be calculated within a short time. Finally, in most current clinical prediction models, drug factors have not been sufficiently considered in development of clinical prediction models, which may limit the application of their findings. Our model incorporates drug factors making it more comprehensive and accurate.

Nonetheless, this study had some limitations. First, the sample size was small, which may increase the possibility of type II errors. In addition, only variables subjected to univariate analysis with *P* < 0.05 were selected for Cox analysis, which to some extent eliminated some risk factors that affect death. Therefore, a study with larger sample size should be conducted to confirm our findings. Second, although the nomogram was subjected to extensive internal validation using bootstrap testing, its performance in other HD patients remain to be clarified. Thus, external assessment should be conducted in wider HD populations. Finally, the eGFR at HD initiation are significantly different according to the used various eGFR equations [31], in this study, the GFR was estimated using the CKD EPI-equation which may result in a lower eGFR value than Cockcroft-Gault equation and MDRD equation at the HD initiation [31]. In addition, the use of calculated GFR instead of measured GFR by radionuclide imaging may not reflect true GFR levels in these patients, therefore the assessment of baseline GFR at the HD initiation may be biased to some extent, and whether baseline GFR is one of the factors affecting the 6-months survival rate in HD patients requires further clinical verification in the future.

## Conclusions

This study developed and validated a nomogram with good accuracy for predicting the 6-months survival of HD patients. Scores were developed for identifying HD patients who were at high risk of death. Based on our results, it will be interesting to evaluate whether close monitoring of blood pressure and blood K levels (or alternatively, replacing ACEi/ARB with other antihypertensive therapies), avoiding excessive ultrafiltration rates, and increasing the use of loop diuretics will reduce all-cause mortality risk of incident HD patients in the first six months after HD initiation. Our results may suggest that it is possible to improve the survival rate of HD patients by reducing the use of temporary dialysis catheter, and planned establishment of AVF prior to dialysis. In summary, the nomogram may help clinicians better formulate treatment and management measures, including disease monitoring, drug selection, and dialysis access selection for HD patients.

## Data Availability

The data used in this study are available from the corresponding author upon request.
